# A novel min-cost flow method for estimating transcript expression with RNA-Seq

**DOI:** 10.1186/1471-2105-14-S5-S15

**Published:** 2013-04-10

**Authors:** Alexandru I Tomescu, Anna Kuosmanen, Romeo Rizzi, Veli Mäkinen

**Affiliations:** 1 Helsinki Institute for Information Technology HIIT, Department of Computer Science, University of Helsinki, Finland; 2 Department of Computer Science, University of Verona, Italy

## Abstract

**Background:**

Through transcription and alternative splicing, a gene can be transcribed into different RNA sequences (isoforms), depending on the individual, on the tissue the cell is in, or in response to some stimuli. Recent RNA-Seq technology allows for new high-throughput ways for isoform identification and quantification based on short reads, and various methods have been put forward for this non-trivial problem.

**Results:**

In this paper we propose a novel radically different method based on minimum-cost network flows. This has a two-fold advantage: on the one hand, it translates the problem as an established one in the field of network flows, which can be solved in polynomial time, with different existing solvers; on the other hand, it is general enough to encompass many of the previous proposals under the least sum of squares model. Our method works as follows: in order to find the transcripts which best explain, under a given fitness model, a splicing graph resulting from an RNA-Seq experiment, we find a min-cost flow in an offset flow network, under an equivalent cost model. Under very weak assumptions on the fitness model, the optimal flow can be computed in polynomial time. Parsimoniously splitting the flow back into few path transcripts can be done with any of the heuristics and approximations available from the theory of network flows. In the present implementation, we choose the simple strategy of repeatedly removing the heaviest path.

**Conclusions:**

We proposed a new very general method based on network flows for a multiassembly problem arising from isoform identification and quantification with RNA-Seq. Experimental results on prediction accuracy show that our method is very competitive with popular tools such as Cufflinks and IsoLasso. Our tool, called Traph (Transcrips in gRAPHs), is available at: http://www.cs.helsinki.fi/gsa/traph/.

## Background

Recent RNA-Seq technology [1,2] opened a new high-throughput, low cost way for isoform identification and quantification, leading to new understanding of gene regulation in development and disease (e.g., [3]). In an RNA-Seq experiment a set of short reads is produced from mRNA transcripts. The difficulty in assembling these short reads into the transcripts from which they were sampled is non-trivial due to the fact that the transcripts (isoforms) may share exons. As a result, all methods for solving this problem rely on an explicit or implicit graph model. The nodes represent individual reads (overlap graph [4]), or contiguous stretches of DNA uninterrupted by spliced reads (splicing graph [5-7], connectivity graph [8-10]), while the edges are derived from overlaps or from spliced read alignments. Each node and edge has an associated observed coverage, and the problem of isoform identification and quantification is seen as separating the coverage of the graph into individual path components, under different models. Furthermore, this problem was also coined under the broad name 'Multiassembly Problem' [11], a hint that it can arise not only with RNA-Seq data, but also in other biological settings, such as assembling metagenomics reads [12].

Except for Cufflinks [4], all tools mentioned above rely on some optimization engine, whose solving is generally difficult. IsoInfer/IsoLasso [8,9], SLIDE [7], Scripture [10], and CLIIQ [6] exhaustively enumerate all possible candidate paths. For efficiency reasons, each has some restrictions on what a valid candidate path might be, and for each candidate isoform, they define a fitness function. IsoInfer/IsoLasso and SLIDE use a least sum of squares fitness function; IsoLasso and SLIDE both add different shrinkage terms to the fitness function in order to favor isoforms with fewer transcripts, which is computed with a modified LASSO algorithm, or a quadratic program; CLIIQ uses a least sum of absolute differences fitness function, solved by a linear integer program. Cufflinks avoids the problem of exhaustively enumerating all possible paths by returning a minimum path cover, and then assigning expression levels to each path in this cover based on a statistical model. Incidentally, note that computing a minimum path cover (in an acyclic digraph) is done by computing a maximum matching, which can be easily reduced to a flow problem. However, such reduction solves a different (implicitly defined) optimization problem that can be considered as a consensus model in the literature [6-10], mostly because the fitting of expression levels is separated in the process.

### Our contribution

In this paper we propose a radically different and very general method relying on the established field of minimum-cost network flow problems [13]. This will not only provide a simple method and a fast polynomial time algorithm for solving it (as opposed to exhaustively enumerating all possible candidate paths, and then solving a quadratic/integer linear program for evaluating the fitness of each candidate isoform), but it can also lean on the ample literature on splitting a (min-cost) flow into paths, e.g., [14-17].

As in the case of the other tools, our method assumes that a splicing graph has been built for each gene. Each node of the graph corresponds to a stretch of DNA uninterrupted by any spliced read alignment; such sequences are called *segments *in [9], but for simplicity we just call them *exons*. Each edge of the graph corresponds to two exons consecutive in some transcript, that is, to some spliced read whose prefix aligns to the suffix of one exon, and whose suffix aligns to the prefix of another exon. Observe that such a graph can be seen as a directed acyclic graph (DAG, for short), the direction of the edges being according to the absolute position of the exons in the genome. For each exon *v *we can deduct its coverage *cov*(*v*) as the total number of reads aligned to the exon divided by the exon length, and the coverage *cov*(*u, v*) of an edge (*u, v*) as the total number of reads split aligned to that junction between exons *u *and *v*. An mRNA transcript candidate thus becomes a path from some source node to some sink node. The requirement that the transcripts start in a source node and end in a sink node is no restriction, as we can add dummy source/sink nodes as in-/out-neighbors to the nodes where we have indication that some transcript might start/end. Indeed, our splicing graph creation tool uses splicing alignments and coverage information to discover such start/end nodes and accordingly indicates them to our tool.

In order to define a fitness function in the broadest possible terms, let us assume that for each node *v *and edge (*u, v*) of the graph we have convex cost functions $f_{v},f_{uv}:\mathbb{R}\to \mathbb{R}$ modeling how close that node and edge must be explained by the candidate isoform. Then, we can state the problem of isoform identification and quantification as following problem.

**Problem 1 (UTEC) ***Given a splicing DAG G *= (*V, E*) *with coverage values cov*(*v*) *and cov*(*u, v*), *and cost functions f_v_*(·) *and f_uv_*(*·*), *for all v *∈ *V and *(*u, v*) ∈ *E, the *Unannotated Transcript Expression Cover *problem is to find a tuple  P of paths from the sources of G to the sinks of G, with an estimated expression level e*(*P*) *for each path P∈P, which minimize*

$$\text{su}{\text{m}}_{-}\text{err}\left(G,\;P\right):=\underset{v\in V}{\sum }f_{v}\left(\left|cov\left(v\right)-\underset{P\in P:v\;\in \;P}{\sum }e\left(P\right)\right|\right)+\underset{\left(u,v\right)\;\in \;E}{\sum }f_{uv}\left(\left|cov\left(u,\;v\right)-\underset{P\in P\;:\;\left(u,v\right)\;\in \;P}{\sum }e\left(P\right)\right|\right).$$

For example, if for all nodes *v *and edges (*u, v*), *f_v_*(*x*) = *x, f_uv_*(*x*) = *x*, then we have a least sum of absolute differences model as in CLIIQ. If *f_u_*(*x*) = *x*^2^, *f_uv_*(*x*) = *x*^2^, then we have a least sum of squares model as in IsoInfer/IsoLasso and SLIDE; this is the model which we also use in the implementation reported in this paper. Another cost function, suggested by [18], is $f_{v}\left(x\right)=x/\sqrt{cov\left(v\right)}$, $f_{uv}\left(x\right)=x/\sqrt{cov\left(u,v\right)}$ for all nodes *v *and edges (*u, v*). Observe that many of the other biological assumptions of the other tools can be incorporated in the model of Problem UTEC.

We will show that Problem UTEC can be solved in polynomial time, by a reduction to a min-cost flow problem with convex cost functions. We will argue that finding the optimal tuple of paths explaining the graph is equivalent to finding the optimal flow in an offset flow network. Moreover, any splitting of this optimal flow into paths attains the minimum of Problem UTEC. In the same way as some of the other tools try to limit the number of paths explaining a splicing graph by a LASSO approach, we can rely on established methods for splitting any flow into few paths (e.g., [14-17]). In this paper, we employ only the simple linear-time heuristic of repeatedly removing the heaviest path, see e.g., [15].

We give experimental results to study how well the predictions match the ground-truth on simulated data, and how well it fares on real-data, compared to Cufflinks [4] and IsoLasso [9]; our method is very competitive, providing in many cases better precision and recall. We expect our lead to be even greater once we incorporate paired-end read information.

## Methods

We begin by recalling the basic notions of flow and of a min-cost flow problem, and refer to the excellent monograph [13] for further details. A *flow network *(or simply *network*) is a tuple *N *= (*G, b, q*), where *G *= (*V, E*) is a directed graph, *b *is a function assigning a *capacity $b_{uv}\in \mathbb{N}$*to every arc (*u, v*) ∈ *E*, and *q *is a function assigning an *exogenous flow *$q_{v}\in \mathbb{N}$ to every node *v *∈ *V*, such that $\sum_{v\in V}q_{v}=0$. We say that a function *x *assigning to every arc (*u, v*) ∈ *E *a number$x_{uv}\in \mathbb{N}$ is a *flow *over the network *N*, if the following two conditions are satisfied:

1. 0 ≤ *x_uv _*≤ *b_uv_*, for every (*u, v*) ∈ *E*,

2. $\underset{u\in V}{\sum }x_{vu}-\underset{u\in V}{\sum }x_{uv}=q_{v}$, for every *v *∈ *V*,

In a min-cost flow problem, one is additionally given flow cost functions *c_uv_*(·), for every arc (*u, v*) ∈ *E*, and is required to find a flow which minimizes:

$$\underset{\left(u,v\right)\in E}{\sum }c_{uv}\left(x_{uv}\right).$$

It is well-known that, under the assumption that all the flow cost functions *c_uv_*(·) are convex, a min-cost flow can be found in polynomial time [19] (see also [20] for the real-valued flow case).

### The reduction to a min-cost flow problem

We will model Problem UTEC as a min-cost flow problem, thus showing that it can be solved in polynomial time. First, we argue that it can be transformed into the following equivalent problem, where the input exon chaining graph has measured coverages only on arcs.

**Problem 2 (UTEJC) ***Given a splicing DAG G *= (*V, E*) *with coverage values cov*(*u, v*), *and cost functions f_uv_*(·), *for all *(*u, v*) ∈ *E, the *Unannotated Transcript Expression Junction Cover *problem is to find a tuple  P of paths from the sources of G to the sinks of G with an estimated expression level e*(*P*) *for each path P∈P, which minimize*

$$\underset{\left(u,v\right)\;\in E}{\sum }f_{uv}\left(\left|cov\left(u,v\right)-\underset{P\in P:\left(u,v\right)\in P}{\sum }e\left(P\right)\right|\right).$$

Given an input *G *= (*V, E*) for Problem UTEC, we construct an input for Problem UTEJC by replacing every node *v *∈ *V *with two new nodes, *v_in _*and *v_out_*, and an arc (*v_in_, v_out_*), with *cov*(*v_in_, v_out_*) = *cov*(*v*), and $f_{v_{in}v_{out}}\left(x\right)=f_{v}\left(x\right)$. Furthermore, for every arc (*u, v*) ∈ *E*, we replace arc (*u, v*) with the arc (*u_out_, v_in_*), with the same coverage as (*u, v*). It is immediate that optimal solutions for *G *to Problem UTEC are in bijection with the optimal solutions for the transformed graph to Problem UTEJC.

To solve Problem UTEJC, we build an auxiliary offset network with convex costs of the form *c_uv_*(*x*) = *f_uv_*(*x*). An optimal flow for this network will model the offsets (positive or negative) between the measured coverages of the exon chaining graph and their actual expression levels in an optimal solution. Then, we argue that a min-cost flow on this network naturally induces a solution for the UTEJC problem.

Onwards, we denote by $N_{G}^{+}\left(v\right)$ the set of *out-neighbors *of *v *in the directed graph *G*, that is, the set {*w *: (*v, w*) ∈ *E*(*G*)}. Similarly, we denote by $N_{G}^{-}\left(v\right)$ the set of *in-neighbors *of *v *in the directed graph *G*, that is, the set {*u *: (*u, v*) ∈ *E*(*G*)}. When *G *is clear from the context, we will skip it as subscript.

**Figure 1:**
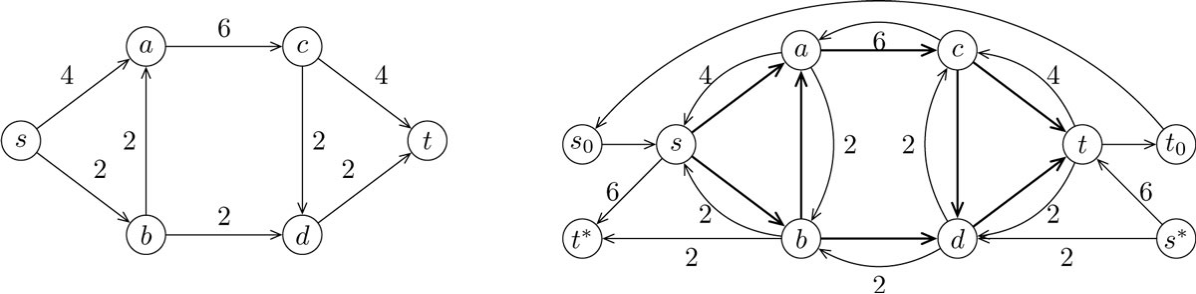
**Example of an offset network**. An input *G *to Problem UTEJC (a), and the offset network G* (b); arcs are labeled with their capacity, unlabeled arcs having infinite capacity

Given a splicing DAG *G *with coverage values *cov*(*u, v*), and cost functions *f_uv_*, for all (*u, v*) ∈ *E*, we construct the offset network N* = (G*, b, q) with cost function *c*, as follows (see Figure 1 for an example):

1. we add to G* all nodes and edges of *G*, together with

(a) a new source *s*_0 _and a new sink *t*_0 _with $q_{s_{0}}:=q_{t_{0}}:=0$,

(b) arcs (*s*_0_, *s*), for every source *s *of *G*, and arcs (*t, t*_0_) for every sink *t *of *G*, each with infinite capacity and null cost function,

(c) arc (*t*_0_, *s*_0_) with infinite capacity and null cost function,

(d) nodes s* and t*, with initial exogenous flow $q_{s^{*}}:=q_{t^{*}}:=0$;

2. for every arc (*u, v*) ∈ *E*(*G*),

(a) *b_uv _*:= ∞, *c_uv_*(*x*) : = *f_uv_*(*x*),

(b) we add the reverse arc (*v, u*) to G* with *b_vu _*:= *cov*(*u, v*), *c_vu_*(*x*) : = *f_uv_*(*x*);

3. for every *v *∈ *V*(*G*),

(a) its exogenous flow *q_v _*is zero,

(b) if $\sum_{u\in N^{+}\left(v\right)}cov\left(v,\;u\right)-\sum_{u\in N^{-}\left(v\right)}cov\left(u,\;v\right)>0$, we add arc (v, t*) to G* where:

i. $\;b_{vt*}:=\sum_{u\in N^{+}\left(v\right)}cov\left(v,\;u\right)-\sum_{u\in N^{-}\left(v\right)}cov\left(u,\;v\right),\;c_{vt*}\left(x\right)\;:=0,$

ii we update $q_{t*}:=q_{t*}+\sum_{u\in N^{-}\left(v\right)}cov\left(u,v\right)-\sum_{u\in N^{+}\left(v\right)}cov\left(v,u\right)$;

(c) if $\sum_{u\in N^{+}\left(v\right)}cov\left(v,\;u\right)-\sum_{u\in N^{-}\left(v\right)}cov\left(u,\;v\right)<0$, we add arc (s*, *v*) to G* where:

i. $b_{s*v}\;:=\sum_{u\in N^{-}\left(v\right)}cov\left(v,u\right)-\sum_{u\in N^{+}\left(v\right)}cov\left(u,v\right),\;c_{s*v}\left(x\right)\;:=0$,

ii. we update $q_{s^{*}}:=q_{s^{*}}+\sum_{u\in N^{-}\left(v\right)}cov\left(v,\;u\right)-\sum_{u\in N^{+}\left(v\right)}cov\left(u,\;v\right)$.

The next lemma shows that there exists a min-cost flow x* on N*.

**Lemma 1 ***Given a digraph G with arc coverages cov*(·,·), the offset network N* = (G*, *b, q*) *constructed as above is a flow network, i.e*., $\sum_{v\in V\left(G*\right)}q_{v}=0$.

**Proof: **Since *q_v _*= 0, for all *v *∈ *V *(G*) \ {s*, t*}, it remains to show that $q_{s^{*}}+q_{t^{*}}=0$. Indeed,

$$\begin{array}{lll} q_{s^{*}}+q_{t^{*}} & =\underset{v\in V\left(G\right)}{\sum }\left(\underset{u\in N_{G}^{-}\left(v\right)}{\sum }cov\left(u,v\right)-\underset{u\in N_{G}^{+}\left(v\right)}{\sum }cov\left(v,u\right)\right)\; & \text{(1)}\\ & =\underset{v\in V\left(G\right)}{\sum }\underset{u\in N_{G}^{-}\left(v\right)}{\sum }cov\left(u,v\right)-\underset{v\in V\left(G\right)}{\sum }\underset{u\in N_{G}^{+}\left(v\right)}{\sum }cov\left(v,u\right)\; & \text{(2)}\\ & =\underset{\left(u,v\right)\in E\left(G\right)}{\sum }cov\left(u,v\right)-\underset{\left(v,u\right)\in E\left(G\right)}{\sum }cov\left(v,u\right)=0\; & \text{(3)}\\ & \; & \text{(4)} \end{array}$$

   □

From such a flow x*, we construct the function *x *on the edges *G *as follows. First, observe that for every arc (*u, v*) ∈ *E*(*G*), at most one of $x_{uv}^{*}$ or $x_{vu}^{*}$is nonnull. Indeed, if this were not the case, then a flow y* which coincides with x*, except for $y_{uv}^{*}:=x_{uv}^{*}-\text{min}\left(x_{uv}^{*},x_{vu}^{*}\right)$ and $y_{vu}^{*}:=x_{vu}^{*}-\text{min}\left(x_{uv}^{*},x_{vu}^{*}\right),$ is also a flow on N* and has a strictly smaller cost than x*, contradicting the fact that x* is of minimum cost. Then, for each arc (*u, v*) ∈ *E*(*G*) we set:

$$x_{uv}:=cov\left(u,v\right)+x_{uv}^{*}-x_{vu}^{*}.$$

### From a flow to a set of paths

Theorem 1 below will argue that the above defined function *x *is a flow on *G *(points (1), (2)), whose arcs we consider to have unbounded capacities and whose nodes, apart from the sources and sinks, have exogenous flow 0. It is a well-known result from classical network flow theory that such a flow can be decomposed into paths, that is, there exist paths *P*_1 _, . . . , *P_t _*from the sources of *G *to the sinks of *G*, having weights *w*_1_, . . . , *w_t_*, respectively, such that, for every (*u, v*) ∈ *E*(*G*) we have

$$x_{uv}=\underset{i:\left(u,v\right)\text{belongs}\text{to}P_{i}}{\sum }w_{i}.$$

Moreover, a decomposition of *x *into at most |*E*(*G*)| paths always exists and can be found in time |*V *(*G*)| · *E*(*G*). Theorem 1 also shows that the paths of any decomposition of *x *are an optimal solution for *G *to Problem UTEJC (point (3)).

**Theorem 1 **Given an optimal flow x* on G*, the function × on G just constructed satisfies the properties, where S denotes the set of sources of G, and T denotes the set of sinks of G:

*(1) for all v *∈ *V *(*G*) \ (*S *∪ *T*), *$\sum_{u\in N_{G}^{-}\left(v\right)}x_{uv}=\sum_{u\in N_{G}^{+}\left(v\right)}x_{vu}$;*

*(2) *$\sum_{s\in S}\sum_{v\in N_{G}^{+}\left(s\right)}x_{sv}=\sum_{t\in T}\sum_{u\in N_{G}^{-}\left(t\right)}x_{ut}$

*(3) any decomposition of × into paths attains the minimum of the objective function of Problem UTEJC, on input G*.

**Proof: **(1): Let *v *∈ *V *(*G*) \ (*S *∪ *T*); by the definition of *x*, we can write

$$\begin{array}{ll} \sum_{u\in N_{G}^{-}(v)}x_{uv} & -\sum_{u\in N_{G}^{+}(v)}x_{vu}=\sum_{N_{G}^{-}(v)}(cov(u,\;v)+x_{uv}^{*}-x_{vu}^{*})-\sum_{u\in N_{G}^{+}(v)}(cov(v,\;u)+x_{vu}^{*}-x_{uv}^{*})\\ & =\;\sum_{u\in N_{G}^{-}(v)}cov(u,\;v)-\sum_{N_{G}^{+}(v,\;u}cov(v,\;u)+\\ & +\;\sum_{u\in N_{G}^{-}(v)}x_{uv}^{*}+\sum_{u\in N_{G}^{+}(v)}x_{uv}^{*}-\sum_{u\in N_{G}^{-}(v)}x_{vu}^{*}-\sum_{u\in N_{G}^{+}(v)}x_{vu}^{*}\\ & =\;\sum_{u\in N_{G}^{-}(v)}cov(u,\;v)-\sum_{u\in N_{G}^{+}(v)}cov(v,\;u)+\sum_{u\in N_{G}^{-}(v)\cup N_{G}^{+}(v)}x_{uv}^{*}-\sum_{u\in N_{G}^{-}(v)\cup N_{G}^{+}(v)}x_{vu}^{*}. \end{array}$$

Observe that for all edges entering t* (exiting s*), their flow equals their capacity, as we have adjusted the exogenous flow of t* (of s*) at point 3.(b)ii. (and 3.(c)ii.) in the construction of G*. We distinguish three cases.

First, if $\sum_{u\in N_{G}^{-}\left(v\right)\cup N_{G}^{+}\left(v\right)}x_{uv}^{*}-\sum_{u\in N_{G}^{-}\left(v\right)\cup N_{G}^{+}\left(v\right)}x_{vu}^{*}>0$, then $\sum_{u\in N_{G}^{-}\left(v\right)\cup N_{G}^{+}\left(v\right)}x_{uv}^{*}-\sum_{u\in N_{G}^{-}\left(v\right)\cup N_{G}^{+}\left(v\right)}x_{vu}^{*}=x_{vt^{*}}^{*}$. Since the flow x* uses the arc (v, t*) with its maximum capacity, we have that $x_{vt^{*}}^{*}=b_{vt^{*}}=\sum_{u\in N_{G}^{+}\left(v\right)}cov\left(v,u\right)-\sum_{u\in N_{G}^{-}\left(v\right)}cov\left(u,v\right)$, which shows that $\sum_{u\in N_{G}^{-}\left(v\right)}x_{uv}-\sum_{u\in N_{G}^{+}\left(v\right)}x_{vu}=0$, proving the claim.

Second, if $\sum_{u\in N_{G}^{-}\left(v\right)\cup N_{G}^{+}\left(v\right)}x_{uv}^{*}-\sum_{u\in N_{G}^{-}\left(v\right)\cup N_{G}^{+}\left(v\right)}x_{vu}^{*}<0$, then $\sum_{u\in N_{G}^{-}\left(v\right)\cup N_{G}^{+}\left(v\right)}x_{uv}^{*}-\sum_{u\in N_{G}^{-}\left(v\right)\cup N_{G}^{+}\left(v\right)}x_{vu}^{*}-=-x_{s^{*}v}^{*}$. Since the flow x* uses the arc (s*, *v*) with its maximum capacity, we have that $x_{s*v}^{*}=b_{s*v}=\sum_{u\in N_{G}^{-}\left(v\right)}cov\left(v,u\right)-\sum_{u\in N_{G}^{+}\left(v\right)}cov\left(u,v\right)$, which again proves the claim.

Finally, if $\sum_{u\in N_{G}^{-}\left(v\right)\cup N_{G}^{+}\left(v\right)}x_{uv}^{*}-\sum_{u\in N_{G}^{-}\left(v\right)\cup N_{G}^{+}\left(v\right)}x_{vu}^{*}=0,$ then, by construction there is no edge between *v *and t* or s*, implying, again by construction, that $\sum_{u\in N_{G}^{-}\left(v\right)}cov\left(u,v\right)=\sum_{u\in N_{G}^{+}\left(v\right)}cov\left(v,u\right)$, from which the claim follows.

(2): From the definition of *x*, we have

(1) $$\underset{s\in S}{\sum }\underset{v\in N_{G}^{+}\left(s\right)}{\sum }x_{sv}=\underset{s\in S}{\sum }\left(\underset{v\in N_{G}^{+}\left(s\right)}{\sum }\left(cov\left(s,v\right)+x_{sv}^{*}-x_{vs}^{*}\right)\right)$$

(2) $$\;=\underset{s\in S}{\sum }\left(\underset{v\in N_{G}^{+}\left(s\right)}{\sum }cov\left(s,v\right)+\underset{v\in N_{G}^{+}\left(s\right)}{\sum }x_{sv}^{*}-\underset{v\in N_{G}^{+}\left(s\right)}{\sum }x_{vs}^{*}\right)$$

By construction, since *q_s _*= 0 for all *s *∈ *S*, we have $x_{st^{*}}^{*}+\sum_{v\in N_{G}^{+}\left(s\right)}x_{sv}^{*}=\sum_{v\in N_{G}^{+}\left(s\right)}x_{vs}^{*}+x_{s_{0}s}^{*}$. Therefore, $\sum_{v\in N_{G}^{+}\left(s\right)}x_{sv}^{*}-\sum_{v\in N_{G}^{+}\left(s\right)}x_{vs}^{*}=x_{s_{0}s}^{*}-x_{st^{*}}^{*}=x_{s_{0}s}^{*}-b_{st^{*}}=x_{s_{0}s}^{*}-\sum_{v\in N_{G}^{+}\left(s\right)}cov\left(s,v\right)$. Plugging this into (2), we obtain

(3) $$\underset{s\in S}{\sum }\underset{v\in N_{G}^{+}\left(s\right)}{\sum }x_{sv}=\underset{s\in S}{\sum }x_{s_{0}s}^{*}=x_{t_{0}s_{0}}^{*}.$$

Similarly,

(4) $$\underset{t\in T}{\sum }\underset{u\in N_{G}^{-}\left(t\right)}{\sum }x_{ut}=\underset{t\in T}{\sum }\left(\underset{u\in N_{G}^{-}\left(t\right)}{\sum }\left(cov\left(u,t\right)+x_{ut}^{*}-x_{tu}^{*}\right)\right)$$

(5) $$\;=\underset{t\in T}{\sum }\left(\underset{u\in N_{G}^{-}\left(t\right)}{\sum }cov\left(u,t\right)+\underset{u\in N_{G}^{-}\left(t\right)}{\sum }x_{ut}^{*}-\underset{u\in N_{G}^{-}\left(t\right)}{\sum }x_{tu}^{*}\right)$$

By construction, since *q_t _*= 0 for all *t *∈ *T*, we have $x_{s^{*}t}^{*}+\sum_{u\in N_{G}^{-}\left(t\right)}x_{ut}^{*}=x_{tt_{0}}^{*}-\sum_{u\in N_{G}^{-}\left(t\right)}x_{tu}^{*}$. Therefore, $\sum_{u\in N_{G}^{-}\left(t\right)}x_{ut}^{*}-\sum_{u\in N_{G}^{-}\left(t\right)}x_{tu}^{*}=x_{tt_{0}}^{*}-x_{s^{*}t}^{*}=x_{tt_{0}}^{*}-b_{s^{*}t}=x_{tt_{0}}^{*}-\sum_{u\in N_{G}^{-}\left(t\right)}cov\left(u,t\right)$. Plugging this into (5), we prove the claim, since by (3) we get

(6) $$\underset{t\in T}{\sum }\underset{u\in N_{G}^{-}\left(t\right)}{\sum }x_{ut}=\underset{t\in T}{\sum }x_{tt_{0}}^{*}=x_{t_{0}s_{0}}^{*}=\underset{s\in S}{\sum }\underset{v\in N_{G}^{+}\left(s\right)}{\sum }x_{sv}.$$

(3): Since any tuple of paths $P=\left(P_{1},P_{2},\ldots ,P_{k}\right)$ from sources of *G *to sinks of *G*, induces a flow on *G*, where the exogenous flow of all nodes which are not sources nor sinks is zero, and any such flow can be split into paths from sources to sinks, the minimum value of

(7) $$\underset{\left(u,v\right)\in E\left(G\right)}{\sum }f_{uv}\left(\left|cov\left(u,v\right)-\underset{P_{i}\in P.\cdot \left(u,v\right)\in P_{i}}{\sum }e_{i}\right|\right),$$

over all *k*, all *k*-tuples of paths $P=\left(P_{1},P_{2},...,P_{k}\right)$ from a source of *G *to a sink of *G*, and over all expression levels *e_i _*for each *P_i_*, is equal to min*_y _*is a flow on G $\sum_{\left(u,v\right)\in E\left(G\right)}f_{uv}\left(\left|cov\left(u,v\right)-y_{v^{\prime }v}\right|\right)$. Since any flow on *G *induces a flow on G*, and vice versa, the above is equal to

$$\underset{z\;\text{is}\;\text{a}\;\text{flow}\;\text{on}{\;G}^{*}}{\text{min}}\underset{\left(u,v\right)\in E\left(G\right)}{\sum }f_{uv}\left(\left|z_{uv}-z_{vu}\right|\right).$$

Since

(8) $$x^{*}=\underset{z\;\text{is}\;\text{a}\;\text{flow}\;\text{on}{\;G}^{*}}{\text{argmin}}\underset{\left(u,v\right)\in E\left(G\right)}{\sum }f_{uv}\left(z_{uv}\right)+f_{uv}\left(z_{vu}\right),$$

and from minimality, for all arcs (*u, v*) ∈ *E*(*G*), at most one of *z_uv _*or *z_vu _*is non null, we have that x* also attains the minimum in (7), proving the theorem. □

In our implementation we use the min-cost flow engine available in the LEMON Graph Library [21]. If no engine for arbitrary convex cost functions is available, or, more generally, if the cost functions themselves happen not to be convex, one can approximate any cost function with piecewise constant or convex cost functions: e.g., one can replace an arc (*u, v*) of capacity *b_uv_*, with |*b_uv_*| arcs of capacity 1, such that first arc has cost *f*(1), and the *i*th arc, *i >*1, has cost *f*(*i*) - *f*(*i *- 1) (this reduction is only pseudo-polynomial but reveals quite effective in practice), see [13] for further details.

### Decomposing the min-cost flow into few paths

As already shown by the other tools, we are generally interested in *parsimoniously *explaining an RNA-seq experiment, that is, in finding, among the optimal solutions to Problem UTEC, one with a low number of paths. At a closer analysis it can be seen that any flow on a graph *G *= (*V, E*) can be decomposed into at most |*E*| *- *|*V*| + 2 paths [14]. However, decomposing a flow into a minimum number of paths is an NP-hard problem in the strong sense, even when restricted to DAGs [14,15]. To overcome this limitation, various heuristics and approximations have been put forth, see, e.g., [14-17] and the references therein. The advantage of our method is that once we have obtained the optimal flow, we can apply any of these methods to split the flow into few paths. For simplicity, in this paper we employ the policy of repeatedly removing the heaviest path, see, e.g., [15]: until the network has null flow, we select a path from the sources to the sinks whose minimum flow on its edges is maximum, report it as transcript, and remove it from the flow network.

## Results and discussion

We call our tool Traph (*T*ranscripts in G*raph*s). We compared Traph to the most used isoform prediction tool Cufflinks [4] and with IsoLasso [9]. We also tried to include SLIDE [7] and CLIIQ [6], but we could not make the former work reliably, and for the latter the publicly available version was not yet available. Full experiment data is available at [22].

We should point out from the start that Traph is not yet employing paired-end read information. Nonetheless, the experiments we report (both simulated and real) are with paired-end reads, Cufflinks and IsoLasso having access to the paired-end information. Moreover, since Traph is not yet employing existing gene annotation information, we ran Cufflinks and IsoLasso without annotation. As already mentioned, we use a least sum of squares model. We experimented in the current implementation with other cost functions, as mentioned in the introduction, $f_{z}\left(x\right)=x,f_{z}\left(x\right)=x/\sqrt{cov\left(z\right)}$, or $f_{z}\left(x\right)=x/cov\left(z\right)$, respectively, for all nodes and edges *z*, but they currently give worse results.

### Matching criteria

In order to match the predicted transcripts with the true transcripts, we take into account the DNA sequences but also the expression levels. For each gene, we construct a bipartite graph with the true transcripts $T=\left(T_{1},T_{2},\ldots \right)$ as nodes in one set of the bipartition, and the predicted transcripts $P=\left(P_{1},P_{2},\ldots \right)$ as nodes in the other set of the bipartition. Empty sequences with 0 expression level were added so that both sets of the bipartition had an equal number of nodes.

To define the costs of the edges of this bipartite graph, let us introduce (cf. Normalized Compression Distance [23]) the binary encoding of a true transcript *T *and its expression level *e*(*T*) with respect to a predicted transcript *P *with expression level *e*(*P*)

(9) code(T|P,j)=γ(j)γ(d+1)editsencoded(T,P)γ(f(e(T)-e(P))),

where γ(*x*) = 0^|*bin*(*x*)-1 ^1*bin*(*x*), *bin*(*x*) being the binary encoding of *x >*0, *j *is the index of *P *in the list of predicted transcripts, *d *is the unit cost (Levenshtein) edit distance of *T *and *P*, editsencoded(*T, P*) lists the edits and gaps between edits using 2-bit fixed code for edit type, 2-bit fixed code for substituted/inserted symbol, and γ(*x*+1) for gap (run of identities) of length *x*, and *f*(*x*) is a bijection between {0, 1, -1, 2, -2, . . .} and {1, 2, 3, 4, 5, . . .} defined as *f*(*x*) = 2*x *for *x *> 0 and *f*(*x*) = 2(*-x*) + 1 otherwise.

Then, the edge cost between nodes $T_{i}\in T$ and $P_{j}\in P$ is defined as |code(*T_i _*| *P_j_, j*)| - |γ(*j*)|. The closer to zero this number is, the better the match between true transcript *T_i_*, with true expression level *e*(*T_i_*) and predicted transcript *P_j _*with predicted expression level *e*(*P_j_*). The minimum weight perfect matching was then computed; this gives a one-to-one mapping between true and predicted transcripts, therefore true transcripts can be ordered in the same order as they match predicted transcripts and code for the index, γ(*j*), is no longer required. Let *edit code length *for an edge between *T_i _*and *P_j _*be *|*γ(*d *+ 1) editsencoded(*T_i_, P_j_*)|, where *d *is the edit distance. Let *bitscore *be edit code length divided by |γ(|*T_i_*| + 1) editsencoded(*T_i_, ε*)|; bitscore is asymmetric, and possibly greater than 1 if *ε *would be a better match to *T_i _*than to *P_j_*, but minimum weight perfect matching chose otherwise for global minimality. Let us also call *relative expression level difference *the ratio |*e*(*P_j_*)-*e*(*T_i_*)|/*e*(*T_i_*). Each matched node pair with relative expression level difference and (edit) bitscore under some given thresholds define a true positive event (TP). The other kind of nodes define false positive (FP) and false negative (FN) events depending on which side of the bipartite graph they reside. Prediction efficiency based on precision, recall and F-measure is also employed in [6,9].

### Simulated human data

As in the case of the other tools, we deem that validating against simulated data is a prerequisite, since, in general, on real data, we do not have available ground-truth. We designed the following validation experiment, closely following the approaches in [6,9]. We chose a set of genes at random, and looked up the corresponding annotated transcripts from the Ensembl database. Out of these genes, we selected only those having between 2 and 5 transcripts. In all, we had 29 genes. For each transcript, we simulated reads with the RNASeqReadSimulator [24]. This simulator chooses an expression level at random from lognormal distribution with mean -4 and variance 1. For each gene, it simulated paired-end reads, with fragment length mean 300 and standard deviation 20, as follows: a transcript was chosen randomly using its expression level as distribution weight, while the position of the read within the transcript was chosen uniformly. As argued in the case of IsoLasso [9], various error models can be incorporated in these steps, but we chose to compare the performance of the methods in neutral conditions. We mapped the reads with TopHat [25]: these read mapping results were given as input to the tested prediction software, and to a Python program which we wrote to construct the splicing graphs needed for Traph. Cufflinks and IsoLasso were ran with the default parameters, because the parameters they offer relate to RNA-seq lab protocol, which was not simulated; we could not see changes to other parameters which could be relevant to the prediction. We use FPKM values as expression levels.

We devised two experiment setups. In the first one, which we call single genes, 300, 000 paired-end reads were generated independently from the transcripts of each of the 29 genes, with the already assigned expression levels. They were independently given to TopHat for alignment, and these independent alignment results were fed to each tool. In the second, more realistic experiment, which we call batch mode, the transcripts and their assigned expression levels were combined into one file, and from this file 29 * 300, 000 paired-end reads were simulated. All these reads were fed to TopHat for alignment, and these combined alignment results were fed to the tools. The fragment length mean and standard deviation were passed to the tools, except for Cufflinks in batch mode, when it was able to infer them automatically.

**Figure 2:**
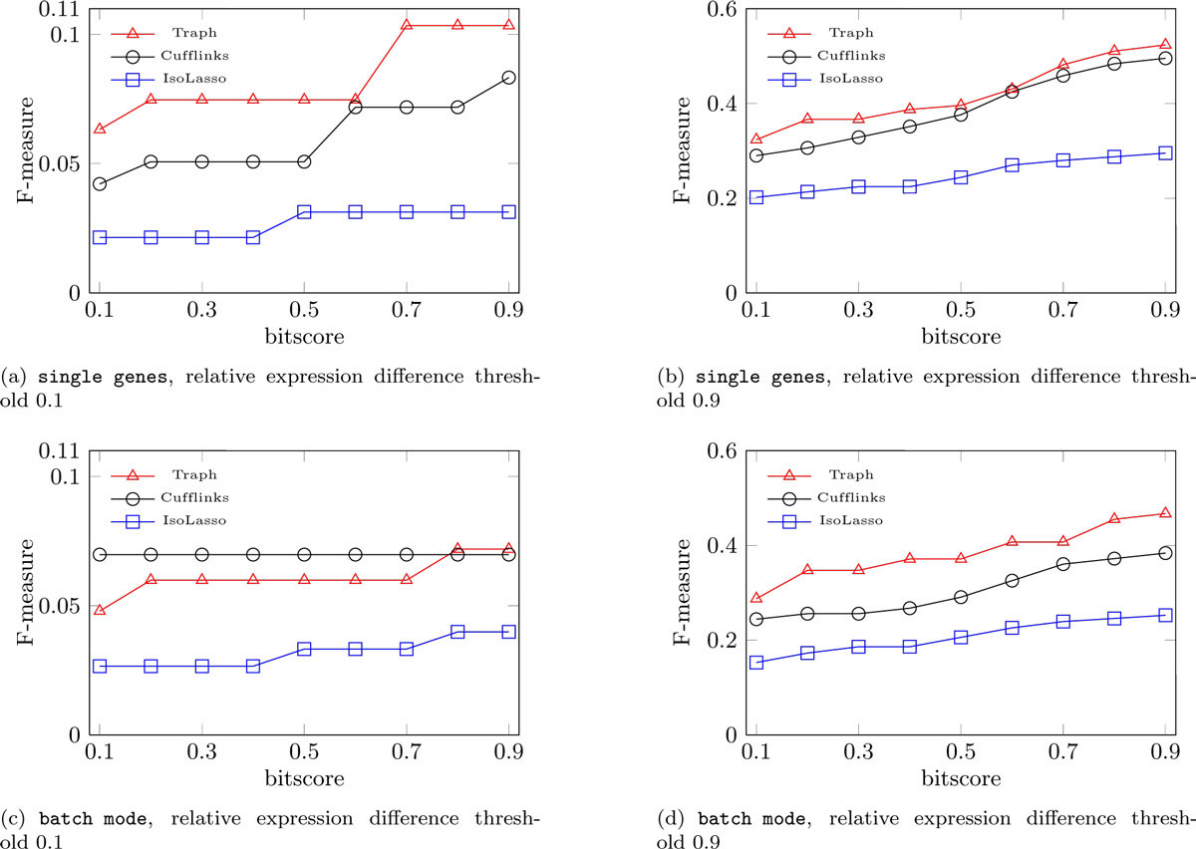
**Performance on simulated data**. Performance of IsoLasso, Cufflinks, and Traph on simulated data: single genes scenario (a), (b); batch mode scenario (c), (d)

Table 1 and Figure 2 show selected validation results. The measures reported are precision = TP/(TP+FP), recall = TP/(TP+FN), and F-measure = 2 * precision * recall/(precision + recall). We selected to depict two relative expression level differences, 0.1 and 0.9, illustrating opposite expression levels matching criteria. In the first, we require that the predicted expression levels be at most 10% different from the true ones, and in the second they can be at most 90% different from the true ones. Even though not yet employing paired-end information, Traph has better F-measure in three out of four scenarios. The lead of Traph is more visible in the batch mode scenario when the predicted expression levels can be at most 90% different from the true ones (Figure 2). This behavior might be due to the upward/downward coverage at the start/end of transcripts, which affects the average coverage Traph is assuming for source/sink nodes (exons). We expect to solve this by giving less weight to such exons in the fitness function. Notice also that out of the false positive transcripts reported by the tools, Cufflinks is reporting 32 transcripts which do not map to the areas of the 29 genes from where reads were simulated, IsoLasso is reporting 150 transcripts outside gene areas, while Traph is reporting only 15.

**Table 1:** Performance of the three tools

	Precision		Recall		F-measure		Avg. run time/gene
	**(0.1, 0.2)**	**(0.9, 0.2)**	**(0.1, 0.2)**	**(0.9, 0.2)**	**(0.1, 0.2)**	**(0.9, 0.2)**	

IsoLasso	0.0183	0.2126	0.0258	0.3109	0.0214	0.2132	25 s
Cufflinks	0.0545	0.3623	0.0482	0.2931	0.0507	0.3060	40 s
Traph	0.0862	0.3729	0.0689	0.3988	0.0747	0.3665	72 s

Performance of the three tools under scrutiny in the single genes scenario; precision, recall and F-measure are computed for (relative expression level difference, bitscore) ∈ {(0.1,0.2),(0.9,0.2)}

### Real human data

We used the same real dataset from the IsoLasso paper [9], Caltech RNA-Seq track from the ENCODE project [GenBank:SRR065504], consisting of 75bp paired-end reads. Out of these reads, we picked the 2,406,339 which mapped to human chromosome 2. We selected the 674 genes where all three tools made some prediction; these genes have 6075 annotated transcripts.

**Figure 3:**
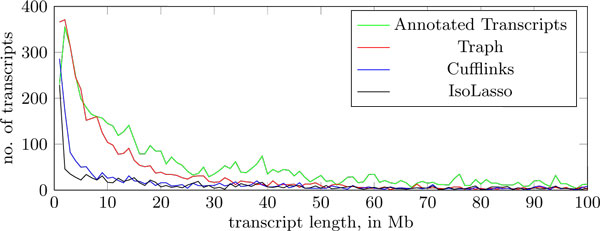
**Results on real human data**. Histogram of the distribution of transcript lengths of the annotation, and of the ones reported by Traph, Cufflinks and IsoLasso

First, we match the transcripts predicted by each tool with the annotated transcripts, employing the same minimum weight perfect matching method introduced before, this time without taking into account expression levels. A true positive is a match selected by the perfect matching with bitscore under 0.2. Traph predicted in total 2685 transcripts for these genes, out of which 244 match the annotation. Cufflinks predicted in total 1796, out of which 349 match the annotation, while IsoLasso predicted 1362, out of which 343 match the annotation. We also include a histogram (Figure 3) of the lengths of the annotated transcripts of these genes, and of the ones reported by Traph, Cufflinks and IsoLasso. Here we round all transcript lengths to the nearest multiple of 1000. We see that the distribution in the case of Traph is closer to the distribution in the case of the annotated transcripts; than the distributions for Cufflinks and IsoLasso.

**Figure 4:**
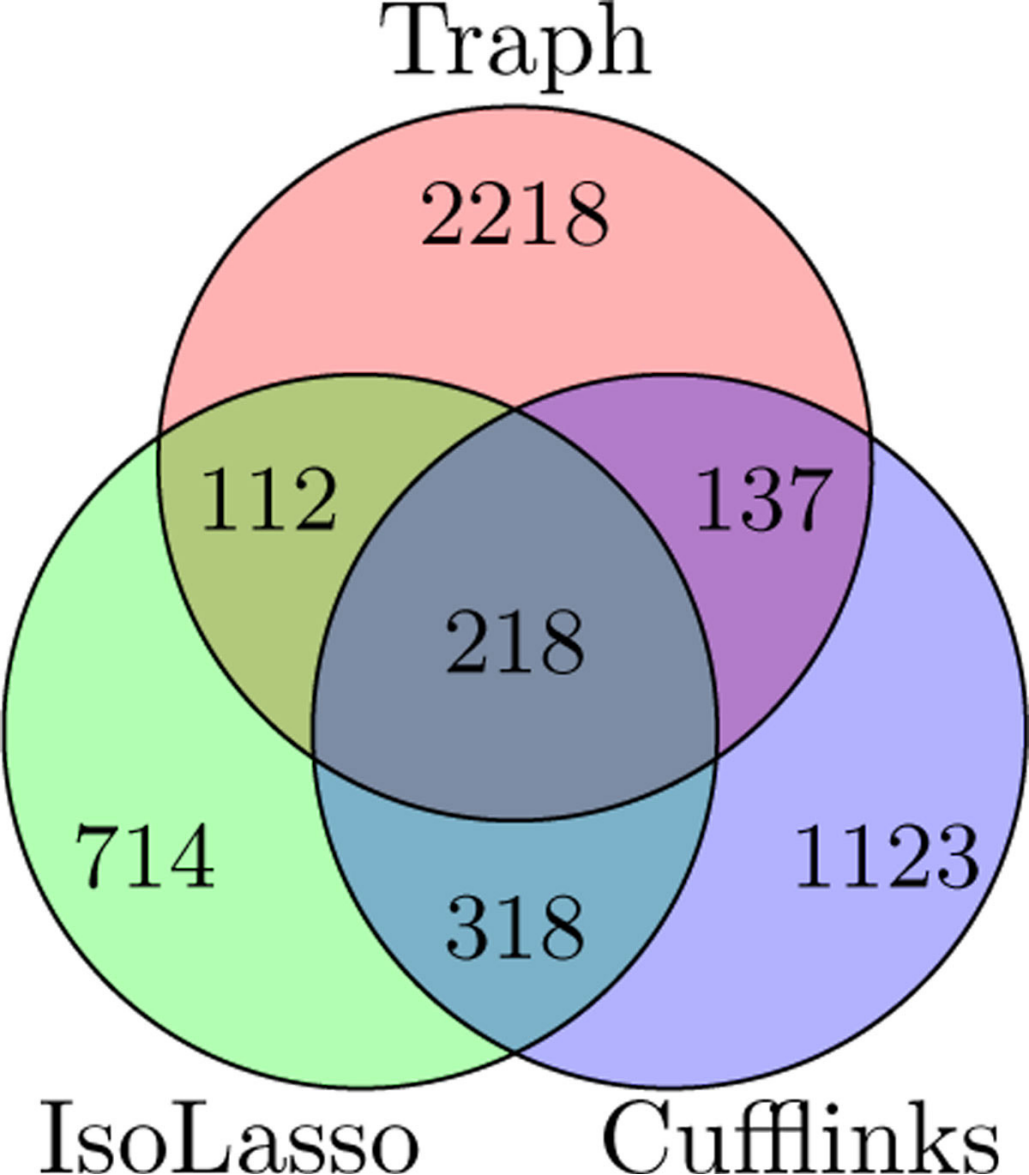
**Results on real human data**. Venn diagram of the intersections of the sets of reported transcripts

Third, we match the transcripts predicted by one software to the transcripts predicted by the other two, employing the same matching method. As in [9], we depict in Figure 4 a more detailed Venn diagram of the intersections between the sets of transcripts reported by the three tools.

### Running times

On the real dataset, Cufflinks finished in 20 min, IsoLasso in 2 min, and Traph in 30 min. We should however stress that for solving the min-cost flow problem and for identifying the transcripts, Traph uses in fact 6 min, the rest of the time being spent by our graph creation tool, which is written in Python. We could not make such a detailed analysis in the case of the other two tools. The running time of our Python script is as well included in the last column of Table 1, where we listed the average running time per gene with simulated reads of each tool.

## Conclusions

All tools for isoform identification and quantification use an explicit or implicit graph model. Resorting to such a representation, the main contribution of this paper consists in a novel, radically different method based on minimum-cost flows, an established problem, for which there exist polynomial-time algorithms and solvers. We implemented this method into our tool Traph. Even though Traph is not using paired-end information at this moment, Traph is competitive with state-of-the-art tools.

This leads us to expect that once we incorporate paired-end read information, the performance of Traph will increase significantly. Note also that in the current implementation, each exon equally contributes to the fitness function, independently of its length; we plan to include exon lengths in the fitness function in a future implementation. We also plan to integrate existing gene annotation into a more refined construction of the splicing graph and into the fitness model. Our method is general enough to easily accommodate other biological assumptions. In order to evaluate the tools against real ground-truth data, we have started a process of acquiring long sequencing reads (PacBio) of the true isoforms of a gene.

## Competing interests

The authors declare that they have no competing interests.

## Authors' contributions

AIT, VM and RR designed the method. VM, AK and AIT designed the experiments. AK evaluated the methods. AIT, VM and AK contributed to the writing. All authors read and approved the final manuscript.

## Declarations

Publication of this article was supported by the Academy of Finland under grant 250345 (CoECGR).

This article has been published as part of *BMC Bioinformatics *Volume 14 Supplement 5, 2013: Proceedings of the Third Annual RECOMB Satellite Workshop on Massively Parallel Sequencing (RECOMB-seq 2013). The full contents of the supplement are available online at http://www.biomedcentral.com/bmcbioinformatics/supplements/14/S5.
